# Modular prevention of heart disease following acute coronary syndrome (ACS) [ISRCTN42984084]

**DOI:** 10.1186/1471-2261-6-26

**Published:** 2006-06-09

**Authors:** Julie Redfern, Elizabeth Ellis, Tom Briffa, SB Freedman

**Affiliations:** 1 School of Physiotherapy, University of Sydney, Sydney, Australia; 2 School of Physiotherapy, Curtin University of Technology, Perth, Australia; 3 Department of Cardiology, Concord Repatriation General Hospital, Sydney, Australia; 4 Faculty of Medicine, University of Sydney, Sydney, Australia

## Abstract

**Background:**

Coronary heart disease (CHD) is a major cause of morbidity and mortality in Australia and it is recommended that all persons with unstable angina (UA) or myocardial infarction (MI) participate in secondary prevention as offered in cardiac rehabilitation (CR) programs. However, the majority of patients do not access standard CR and have higher baseline coronary risk and poorer knowledge of CHD than those persons due to commence CR. The objective of this study is to investigate whether a modular guided self-choice approach to secondary prevention improves coronary risk profile and knowledge in patients who do not access standard CR.

**Methods/Design:**

This randomised controlled trial with one year follow-up will be conducted at a tertiary referral hospital. Participants eligible for but not accessing standard CR will be randomly allocated to either a modular or conventional care group. Modular care will involve participation in individualised modules that involve choice, goal-setting and coaching. Conventional care will involve ongoing heart disease management as directed by the participant's doctors. Both modular and conventional groups will be compared with a contemporary reference group of patients attending CR. Outcomes include measured modifiable risk factors, relative heart disease risk and knowledge of risk factors.

**Discussion:**

We present the rationale and design of a randomised controlled trial testing a modular approachfor the secondary prevention of coronary heart disease following acute coronary syndrome.

## Background

Coronary heart disease (CHD) is a major cause of morbidity and mortality in Australia and is associated with significant cost [1]. Australian guidelines recommend all persons with unstable angina (UA) or myocardial infarction (MI) participate in secondary prevention [2] incorporating risk factor management. Risk factor modification including lifestyle change and the use of vasoprotective medications is an effective means extending overall survival, reducing non-fatal cardiovascular events, decreasing the need for coronary revascularisation, enhancing quality of life (QOL) [3] and improving coronary endothelial function [4,5]. Importantly, CHD is a chronic illness that is best managed when positive health behaviors become integrated into long-term lifestyle habits therefore, strategies are needed to motivate and support patients to consistently follow multidimensional treatment regimens.

Cardiac rehabilitation is a widely recognised form of secondary prevention [6], but participation rates are low (10–30%) [7,8] because of transport difficulties, work and social commitments and a lack of perceived need [9]. Despite clear short-term benefits for attendees, large groups of patients are not benefiting, which presents an opportunity and challenge to improve CHD care [10]. Importantly, there are limited studies reporting the risk profile of patients not accessing formal rehabilitation. One observational Australian study demonstrated that non-participants were at higher risk of recurrent events than patients attending rehabilitation [11]. Similarly, functional impairment has been shown to be the strongest predictor of non-participation in cardiac rehabilitation after coronary artery bypass graft surgery [10]. These findings are of major concern because the patients who stand to benefit the most from secondary prevention are the people least likely to participate.

One pilot study has clinically investigated secondary prevention of CHD using a 'modular' approach and demonstrated enhanced completion rates and cost effectiveness [12]. For the study, a metropolitan cardiac rehabilitation program was offered as individual components, or modules, based on patient need rather than an all-or-nothing exercise-based approach. The pilot study was an important step from standard cardiac rehabilitation however, the modules were still conducted in a group environment and were predominantly based on education without formal goal setting and follow-up. Results of the pilot study suggest it is likely that the development and evaluation of more detailed unique risk factor modules may enhance long-term outcomes.

The inclusion of multidimensional behavior modification techniques such as goal setting and offering a wide range of individually tailored services may enhance compliance with long-term risk factor interventions [13]. For chronic disease, it is important to conceptualise change in a positive light to reduce anxiety about the disease rather than focusing on negative aspects of making a change [14]. Setting of mutually-agreed, realistic and readily identifiable goals with clear time frames helps enhance active patient orientation and motivation and therefore facilitates behavior change [14,15]. Further, the inclusion of a rating of confidence for goal achievement may improve the health professional's insight into a patient's intrinsic motivation to change [14].

Facilitation of collaborative relationships, shared decision-making, offering choice and communication of expectations in a supportive environment helps motivate behavior change and therefore contributes to the disease management process [15,16]. Formation of collaborative relationships decreases patient anxiety and dissatisfaction that is often related to uncertainty and lack of information, explanation and feedback [17]. Studies have demonstrated that encouraging patients to participate in treatment decisions improves health status, QOL [18,19], satisfaction, compliance and treatment outcomes [20]. More specifically, patients with a higher degree of active orientation are more likely to comply with treatment recommendations for BP [16], diabetes and physical activity [21]. Offering choice, in situations where there are several management options based on individual preferences or circumstances is a further method of enhancing active patient orientation [22]. It is also suggested that options offered as a menu of strategies are more successful because it encourages each patient's task to be one of "choosing rather than refuting" [14]. We have chosen the term 'guided self-choice' to describe this model which creates a collaborative encounter, similar to the spirit of behavior counseling [23].

The objective of this study is to investigate whether a modular guided self-choice approach to secondary prevention improves coronary risk profile and knowledge in acute coronary syndrome (ACS) patients who do not access standard cardiac rehabilitation.

## Methods/Design

### Design

This randomised controlled trial with one year follow-up will be conducted at a tertiary referral hospital in Sydney, Australia. Participants not attending standard cardiac rehabilitation will be randomly allocated to either a modular secondary prevention (experimental) or a conventional care (control) group. A third contemporary group of patients attending standard cardiac rehabilitation will also be recruited (reference group). Ethical approval for the study has been granted by both the Central Sydney Area Health Service CRGH Zone and the University of Sydney Human Research Ethics Committees. Written and informed consent will be obtained from all participants prior to commencement.

Independent variables are the identification of individualised risk factor goals and participation in self chosen secondary prevention modules. The dependent variables are assessment of cardiac risk factors, the achievement of risk factor goals, unplanned hospital readmissions, quality of life, relative cardiac risk and knowledge of risk factors. The measurement and recording of experimental variables will be conducted under blind conditions.

### Study population

To establish a recruitment database, a list of all admissions to a single metropolitan tertiary referral hospital in Sydney Australia, with an ACS during a 10 month period, was obtained using diagnostic related codes [24] for UA (F72A, F72B), MI (F41A, F41B, F60A, F60B) and chest pain (F74Z). For all admitted patients, the hospital medical records were reviewed to determine secondary prevention eligibility. Reasons for ineligibility included geography (living outside a 20 km radius of the hospital), insufficient English language to provide informed consent, congestive heart failure, the presence of a severe co-morbidity (eg, end stage renal disease, Parkinson's disease, cancer) and death.

Inclusion criteria for the randomised conventional care and modular groups were:

- No previous attendance at a formal cardiac rehabilitation program including those who refused the initial invitation to participate and those who failed to attend the initial cardiac rehabilitation assessment.

- Diagnosis of ACS in the six months prior to recruitment.

- Ability to understand sufficient English to give written and informed consent.

Inclusion criteria for non-randomised standard rehabilitation group were:

- Commencing attendance at the CRGH cardiac rehabilitation program during the data collection period of the study.

- Diagnosis of ACS in the six months prior to recruitment.

- Ability to understand sufficient English to give written and informed consent.

Exclusion criteria were the same for all three groups in the study and were:

- Clinical diagnosis of uncompensated, severe cardiac failure (Class IV).

- Uncontrolled arrhythmia or angina

- Severe or symptomatic aortic stenosis

- Persistent hypotension (SBP < 90 mmHg)

- Clinical diagnosis of a severe co-existing medical condition that would prevent participation (eg, dementia, a rapidly deteriorating terminal illness or severe or active rheumatoid arthritis)

Once the database of eligible patients is constructed, all patients will be contacted by mail and telephone and asked to volunteer for research investigating heart disease risk factors. Recruitment mail-outs will be conducted in batches of 30 to enable staggered recruitment over approximately six months. Patients will be contacted within two weeks of the mail-out to establish whether they would be prepared to volunteer.

### Group allocation

For the standard cardiac rehabilitation group, participants will be consecutively recruited prior to commencement of outpatient cardiac rehabilitation. Patients who are eligible for but not participating in cardiac rehabilitation will be randomly allocated to either their modular or conventional care group immediately following completion of the baseline assessment. Therefore, the researcher conducting each baseline assessment will be blinded to group allocation. Prior to study commencement, a computer-generated allocation sequence will be constructed using randomly permutated blocks [25]. An individual not involved in the study recruitment, data collection or analysis will produce consecutively numbered sealed opaque envelopes containing each participant's allocation. Following baseline assessment, the researcher will select the next consecutively numbered envelope and open it to reveal the allocated group. They will then notate the unique patient identifier on the front on the participants cover sheet. Therefore, group allocation will be concealed from the researcher conducting baseline assessments.

### Outcome measures

Assessments will be conducted in the hospital consulting rooms or in the participant's home, if they are unable to travel to the hospital. Demographic, subjective and objective information will be obtained during face-to-face assessments at baseline, three and 12 months. Information will include past history, nature of ACS, family history, age, medications and dose, presence of other cardiovascular disease and the nature and date of any revascularisation. In addition, contact details for each participant, their general practitioner and their cardiologist will be documented.

At three and 12 month follow-up, all baseline measures will be repeated during face-to-face assessments by a researcher blinded to group allocation. Additional collected information will include – details of unplanned hospital admissions, medications and doses along with details of any health interventions undertaken by the participant pertaining to their heart health, such as visits to the GP and attendance at community programs. For participants in the standard rehabilitation group, details of participation in the cardiac rehabilitation program will also be recorded such as duration and attendance at exercise and education sessions.

Modifiable risk factors to be measured are serum cholesterol, BP, smoking status, physical activity, overweight and depression. Lipids and substrates will be measured on a fasting blood sample within two weeks of assessment date. Participants will be referred to local pathology clinics by their primary care doctor and will visit the clinics by their own arrangement. Blood samples will be analysed for determination of total cholesterol (TC), low density lipoprotein cholesterol (LDL-C), high density lipoprotein cholesterol (HDL-C) and triglycerides (TG). Repeat blood tests will be carried out at the same pathology clinic wherever possible.

Resting BP will be measured from the brachial artery using an Omron digital automatic BP (Omron Healthcare Co Ltd Model M5, Japan). The automatic BP monitor will be calibrated against a mercury sphygmomanometer prior to study commencement and has been demonstrated to be accurate. The Omron M6 is accurate for BP to within 3 mmHg [26] and fulfills the recommendation criteria of the international protocol when tested against a mercury sphygmomanometer for diastolic and systolic BP readings [27]. For measurement, appropriate cuff size will be selected, each participant will be seated with the right arm supported and two consecutive blood pressure readings will be collected with one minute interval according to previously published guidelines [28].

Smoking status will be initially assessed by analysis of carbon monoxide (CO) levels using a hand-held breath analyzer or CO meter (Bedfont micro smokerlyzer, Scientific Ltd, England) using new mouthpieces (Airmet Scientific EC50MP500, Victoria Australia) for each participant, for infection control purposes. Participants will be expected to blow into the mouthpiece for the 15 seconds and the result are to be recorded in parts per million (ppm). The criteria for recent, within the past 24 hours, smoking will be an expired CO greater than 5 ppm [29]. Smoking status will be confirmed with self report to identify participants who concealed their true smoking status. Participants will be asked whether they had ever smoked, how long since they started or stopped and if they smoked, what is/was the average number of cigarettes smoked per day.

Physical activity levels will be assessed using the International Physical Activity Questionnaire (IPAQ) score [30]. The short, last-seven-days, self-administered format (last revised August 2002) comprises seven questions that seek information about the duration and frequency of vigorous and moderate physical activity as well as rest and walking in the past seven days. The IPAQ was developed as an international measure of physical activity, for use in adults, and has been tested for reliability and validity across 12 countries [31]. For final scoring, responses are divided into three categories, MET-minutes per week, average time spent sitting per day and a categorical level of physical activity. The categorical score is further divided into three levels – insufficiently active, sufficient activity and highly active.

For assessment of depressive mood, the Cardiac Depression Scale (CDS) score will be used [32]. The CDS was developed from the responses of cardiac outpatients and comprises 26 items with subscales relating to sleep, uncertainty, mood, cognition, hopelessness and inactivity. Some items are scored in reverse and raw scores are used for analysis where higher scores indicate depressed mood and a score of greater than 90 represents depressive mood. When assessed for test-retest reliability over two weeks scores were 64.9 ± 23.3 (mean ± SD) and 63.1 ± 23.0 with correlation coefficient of 0.86 and score difference of -1.9 ± 12.2 (mean ± SD). Permission has been obtained to administer the CDS from Professor Hare, Department of Cardiology (Austin & Repatriation Medical Centre, Australia).

For assessment of overweight, body mass index (BMI) will be calculated using the standard equation (BMI = weight kg/height m^2^). Each participant's body weight will be measured when lightly clothed and in bare feet using digital scales (Tanita EB727, Australia) to the nearest 0.1 kg. Height will be measured without shoes using a standiometer (Stature Meter 2 m 1013522, Livingston International Pty Ltd, NSW Australia) to the nearest 0.5 cm and according to best anthropometic practice [33].

Health related quality of life (HRQOL) will be assessed using the SF36 health survey [34], [35]. The SF36 comprises 11 items that ask for the participant's views about their health, how they feel and whether they are able to perform certain activities and has been tested for reliability and validity [34], [35]. A license has been obtained to use the SF36 for data collection from 300 patients on three occasions per participant (License number F1-052203-13860). SF36 scores will be coded, summed and transformed onto a scale from 0 (worst possible health) to 100 (best possible health) using the method described in the user manual [36].

Relative cardiac risk will be calculated using the LIPID score which was developed for use in secondary prevention [37]. An individual's risk score calculation involves aggregating risk point estimates corresponding to the patient's risk factor profile. Aggregate scores provide an indication of the level of risk for a future ACS. The scores are divided as low risk (≤ 4), medium risk (5–6), high risk (7–9) and very high risk (≥ 10) [37]. Further, each participants number of modifiable risk factors will be calculated using the cut points; TC > 4.0 mmol/L, SBP ≥ 140 mmHg, current smoking, physical inactivity using the categorical IPAQ score, BMI ≥ 30 kg/m^2^, known diabetes and a CDS score ≥ 90 for depression.

Each participant's knowledge of their own cardiovascular risk factors will also be assessed at baseline, three and 12 months. Participants will be asked if they can state any of their own modifiable risk factors for heart disease and the number stated will be compared to the each individual's actual number of modifiable risk factors. Participants will then be asked if they are able to state the Australian recommended targets for total cholesterol, BP, physical activity and whether or not smoking is recommended for people with CHD.

### Procedure

#### Conventional care group

Participants randomly allocated to the conventional care group will continue to manage their heart health as directed by their primary care doctor or by their own cognisance. This group will not receive any additional intervention as a result of participation in the study. However, they will be contacted by telephone 2–3 weeks after baseline assessment to confirm their three month assessment appointment. In addition, each participant's general practitioner (GP) and cardiologist will be mailed a courtesy letter following baseline assessment and a risk factor summary with recommendations for discussion regarding long-term risk factor management following the final 12 month assessment.

#### Modular group

Participants randomly allocated to the modular group will manage their heart health by participation in modular guided self-choice management of heart disease. That is, participants will participate in risk factor modules of their choice, with the exception of the mandatory blood cholesterol module, strive to achieve mutually-agreed goals and make choices about risk factor reduction interventions based on their own circumstances. Following baseline assessment, the consultation will be extended by approximately 30 minutes for selection of modules, goal setting and shared decision making about risk factor interventions.

The risk factor modules consist of cholesterol-lowering, BP management, smoking cessation and physical activity. All participants will participate in the cholesterol-lowering module and where applicable, participants can also choose up to two other modules depending on their individual risk factor assessment and personal preference. Therefore, all participants will participate in a minimum of one risk factor module and a maximum of three modules at any one time.

The mandatory cholesterol-lowering module involves pharmacological intervention in consultation with the primary care doctor as well as education and empowerment through information and support about individual and nationally recommended cholesterol levels. The three optional modules involve choice of intervention including; following medical advice; participating in a structured program (individual or group); participating in a home program; or self-help. All risk factor modules will be resourced and delivered separately among the primary care doctor, the local area health service, the local community and the participant. Participants in the modular group will receive a resource package including appropriate information leaflets which contain guidelines and information about the module and contact information for community heart health programs. The leaflets have been developed and pilot tested, for validity, by health professionals and consumers.

For each risk factor module, a three month objective goal will be set based on shared decision-making. The mutually-agreed goal will be based on the each participant's risk factor profile, social factors, commitments and personal preference. Participants will also asked to rate their confidence in achieving the set goal on a visual analogue scale from 1–10. Each risk factor baseline level, mutually agreed goal, time frame and confidence rating will be recorded on the information leaflet.

All participants in the modular group will also be "coached" using a previously published model [38]. The coaching model is a five stage cyclic process and involves assessing understanding, providing education, assertiveness training and reassessment. In this study, the first coaching session will be conducted during the initial face-to-face shared decision making, goal setting session. Participants will then be coached over the telephone on up to four further occasions, depending on goal achievement, during the following three months depending on need. Once each risk factor goal is achieved, no further coaching is required. All coaching will be conducted by a researcher who has been trained by the original coach study coordinator. During each coaching session individual's knowledge of their risk factors and personal goals will be identified and participation in the module will be established and recorded.

Communication between the study coordinator, the participant and health professionals involved and community services will be facilitated where possible. Each participant will also be encouraged to communicate openly about their goals and modules with their local doctor and cardiologist. During the course of the study, participants identified as being clinically depressed, according to their CDS, are to be offered an appointment with a clinical psychologist specialising in heart disease. For each participant, their GP and cardiologist will be sent a comprehensive letter outlining the participant's risk factors, goals and chosen modules. Following the final 12 month assessment, all participants and their doctors will also receive a cardiac risk factor summary and recommendations for discussion regarding long term risk factor management. By facilitating communication, it is anticipated that each participant receives a coordinated multidisciplinary approach to their own risk factor management based on need and situation.

#### Standard rehabilitation group

Participants who volunteer for the standard rehabilitation group will attend the hospital-based outpatient cardiac rehabilitation program. This rehabilitation program includes twice weekly group hospital-based exercise classes for six weeks and weekly education sessions. The exercise program is supervised by a clinical nurse specialist and a physiotherapist. The exercise sessions are generally of 45 minutes duration and include a 10 minute warm up and cool down and approximately 30 minutes of cardiovascular and resistance exercise stations. These stations include leg press, treadmill walking, stair climbing, knee extension weights, stationary cycling and gentle upper limb weights. Each station is conducted for 3 minutes and patients rotate around the room from station to station with a 2 minute rest between stations. Exercise intensity is based on rating of perceived exertion (RPE) at a moderate level (3–4) on the modified RPE scale [39].

The education sessions, include general information about heart disease and associated risk factors, two sessions by a pharmacist about medications, two sessions about dietary advice by dietitian (one theoretical and one practical session), two sessions from a clinical psychologist and one session about exercise by a physiotherapist. Participants will be encouraged to attend two exercise sessions per week and all the educational sessions if possible. There is no transport available for travel to and from the sessions however, parking is available in the hospital car-park at each patient's expense. Similar to the conventional care group, the standard rehabilitation group will not receive any additional intervention as a result of participation in the study. However, all participants will be contacted by telephone 2–3 weeks after baseline assessment to confirm their three month appointment. In addition, each participant's GP and cardiologist will be mailed a courtesy letter following baseline assessment and a risk factor summary with recommendations for discussion regarding long term risk factor management following the final 12 month assessment.

### Sample size

Participant numbers required for each group have been calculated to detect a 0.5 mmol/L reduction in TC with 80% power. The LIPID study of 9014 subjects with CHD reported a mean TC of 5.6 (± 0.7) mmol/L [40] and it has been assumed that a clinically significant change in TC would be 0.5 mmol/L. Therefore, to achieve power of 80% to detect a change of 0.5 of a SD unit, using a two-tailed test with an alpha level of 0.05 and allow for 10% loss to follow-up, it was calculated that 72 participants per group will be recruited.

### Data analysis

Data will be analysed by intention-to-treat using SPSS for Windows (Version 12.01) and will be presented as mean and standard error of the mean or proportions. Differences in outcome measures, between and within groups, will be compared using repeated measures ANOVAs for continuous variables and either χ^2 ^tests or Fishers exact tests, as appropriate, for proportions of categorical variables. Two tailed p values of < 0.05 will be considered significant.

## Discussion

The majority of patients with CHD do not access existing cardiac rehabilitation programs. Testing of alternative models of secondary prevention will potentially provide beneficial programs for more high risk patients following ACS.

We have presented the rationale and design for a randomised controlled trial testing a modular approach to the secondary prevention of CHD. The study is also designed to compare outcomes to benchmarks set by patients participating in standard cardiac rehabilitation. The primary outcomes will be the magnitude of CHD modifiable risk factors and secondary outcomes will be relative CHD risk and knowledge of these factors.

## Competing interests

The author(s) declare that they have no competing interests.

## Authors' contributions

JR, EE, TB and BF were responsible for the design of the study. All authors read and approved the final manuscript.

## Pre-publication history

The pre-publication history for this paper can be accessed here:


